# Involvement of E3 Ligases and Deubiquitinases in the Control of HIF-α Subunit Abundance

**DOI:** 10.3390/cells8060598

**Published:** 2019-06-15

**Authors:** Kateryna Kubaichuk, Thomas Kietzmann

**Affiliations:** Faculty of Biochemistry and Molecular Medicine, Biocenter Oulu, University of Oulu, 90220 Oulu, Finland; Kateryna.Kubaichuk@oulu.fi

**Keywords:** Hypoxia, DUBs, E3 ligases, ubiquitylation, HIF, cancer

## Abstract

The ubiquitin and hypoxia-inducible factor (HIF) pathways are cellular processes involved in the regulation of a variety of cellular functions. Enzymes called ubiquitin E3 ligases perform protein ubiquitylation. The action of these enzymes can be counteracted by another group of enzymes called deubiquitinases (DUBs), which remove ubiquitin from target proteins. The balanced action of these enzymes allows cells to adapt their protein content to a variety of cellular and environmental stress factors, including hypoxia. While hypoxia appears to be a powerful regulator of the ubiquitylation process, much less is known about the impact of DUBs on the HIF system and hypoxia-regulated DUBs. Moreover, hypoxia and DUBs play crucial roles in many diseases, such as cancer. Hence, DUBs are considered to be promising targets for cancer cell-specific treatment. Here, we review the current knowledge about the role DUBs play in the control of HIFs, the regulation of DUBs by hypoxia, and their implication in cancer progression.

## 1. Introduction

Proteins are involved in practically every aspect of cellular functionality. As components of the cytoskeleton, they take part in maintaining structural support: As enzymes they function to catalyze a variety of biochemical reactions, as hormonal and messenger proteins they are involved in signal transduction, and as antibodies they take part in defense reactions. In mammalian cells, the protein turnover rate is rather high: About 30% of newly synthesized proteins are degraded with a half-life of less than 10 min [1]. Therefore, there is no doubt that maintaining protein homeostasis has to be tightly controlled in the cell, as both excessive protein synthesis and degradation can lead to a manifestation of various diseases, including neurodegenerative disorders, viral diseases, and one of the most common leading causes of death worldwide—cancer.

The intracellular degradation of proteins may be achieved in two ways, via lysosomal proteolysis and ubiquitin-mediated proteasomal degradation [2]. Although several lines of evidence have indicated that these two pathways are interlinked, the action of lysosomes is rather broad [2]. In addition to proteins, they also break down various biomolecules from outside and inside the cell (autophagy), including nucleic acids, carbohydrates, and lipids. In contrast to lysosomal degradation, ubiquitin-mediated proteasomal protein degradation is considered to be highly selective and has therefore gained specific interest in cancer therapy. Indeed, the proteasome inhibitors bortezomib, carfilzomib, and ixazomib have been approved for mantle cell lymphoma and multiple myeloma therapy [3,4]. The mechanisms by which proteasomal inhibitors kill cells is rather unspecific and comprises the accumulation of misfolded proteins and reactive oxygen species (ROS), the upregulation of proapoptotic proteins, the stabilization of p53, as well as antiangiogenic actions [3].

Cancers, especially solid cancers, are also characterized by their limited oxygenation (commonly called hypoxia) [5]. To sustain hypoxia, cells initiate an adaptation program where proteins from the hypoxia-inducible transcription factor-α family (HIF) play a key role. Importantly, HIFs themselves are subject to an oxygen-dependent degradation involving ubiquitylation and subsequent proteasomal degradation [6]. Interestingly, the process of ubiquitylation is reversible and carried out by enzymes termed deubiquitinases (DUBs) [7]. While hypoxia appears to be a powerful regulator of the ubiquitylation process, much less is known about hypoxia-regulated DUBs and the impact of DUBs on the HIF system. Therefore, the current review aims to summarize the role DUBs play in hypoxia signaling and cancer progression.

## 2. Ubiquitin-Mediated Proteasomal Degradation

Ubiquitylation is a highly dynamic process, where ubiquitin (Ub) is covalently linked to lysine residues in target proteins. Although ubiquitylation is best known for its role in marking proteins for subsequent degradation by the 26S proteasome, ubiquitin conjugation has many more diverse functions depending on the nature of the ubiquitin modification (see below). At its most simple level, ubiquitylation requires, apart from the target protein Ub as a tagging factor, the sequential action of a Ub-activating (E1), Ub-conjugating (E2), and Ub-ligating (E3) enzyme [7].

Ubiquitin is a small, highly conserved protein with a molecular weight of 8.5 kDa, which is attached either as a monomer or as an isopeptide-linked polymer to the target proteins. The isopeptide bond between Ub and the target is usually formed between the carboxy terminal glycine residue (G76) of ubiquitin and the ε-amino group of a lysine residue in the target [8].

As mentioned before, the action of three enzymes (E1, E2, and E3) is required for successful attachment of ubiquitin to the substrates [9]. First, the E1 enzyme activates ubiquitin by forming a thioester bond between the carboxy terminal glycine residue of ubiquitin and the active site cysteine in E1. Adenosine triphosphate (ATP) is required for this step. Second, activated Ub is transferred from E1 to the active site of one of 30–40 Ub-conjugating (E2) enzymes. In the third step, Ub is transferred with the help of an E3 Ub ligase from the Ub-loaded E2 to a target substrate that is specifically recognized and bound to E3 [10].

**Figure 1:**
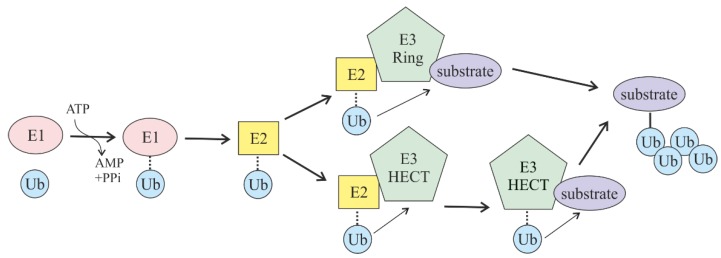
Scheme of protein substrate ubiquitylation. This pathway requires ubiquitin (Ub) and the availability of Ub-activating (E1), Ub-conjugating (E2), and Ub-ligating (E3) enzymes. Two major classes of Ub-ligating enzymes, really interesting new gene (RING)- and homologous to the E6-AP carboxyl terminus (HECT)-domain-containing E3 ligases, are presented.

Taking into account that the number of E3 Ub ligases in a cell is ~650, it is of no surprise that these enzymes are acclaimed as the main specificity factors in the whole ubiquitin-mediated proteasomal degradation pathway. There are two main families of E3 Ub ligases, which differ in the mechanism of ubiquitin transfer: Really interesting new gene (RING)-domain-containing and homologous to the E6-AP carboxyl terminus (HECT)-domain-containing ligases. RING E3 ligases (and the structurally related U-box E3s) are complexes that act as a scaffold to ensure the direct transfer of ubiquitin from E2 to the target lysine in the substrate. In contrast, HECT E3 ligases first transfer Ub from E2 to the active-site cysteine within the HECT domain of E3, and from there it is then transferred to a lysine in the substrate bound to E3. Over 92% of the E3 Ub ligases in the cell are RING-domain-containing E3 ligases, while the rest are HECT and other smaller families of ligases (plant homology domain, zinc finger, and U-box) [11] (Figure 1).

Three different types of ubiquitylations are known: (a) Monoubiquitylation, when only a single molecule of ubiquitin is attached to the target substrate; (b) multi-monoubiquitylation, when several single ubiquitin molecules are attached to the target protein; and (c) polyubiquitylation, when polyubiquitin chains are attached to one or several lysines (K) of the substrate [12]. Monoubiquitylation usually plays the role of a reversible, nonproteolytic signal that is often involved in endocytosis, endosomal sorting, DNA repair, and histone regulation [13,14,15]. Multi-monoubiquitylation is known to be involved in endocytosis and receptor internalization [16,17]. Polyubiquitylation is the most common ubiquitin modification that tags target proteins for degradation, where at least four Ub molecules are required to target substrates to the proteasome [14]. Recently, it has emerged that poly-Ub can have nonproteolytic functions by taking part in DNA repair, transcription regulation, cell division, inflammation, endocytosis, mitophagy, and signaling [18].

**Figure 2:**
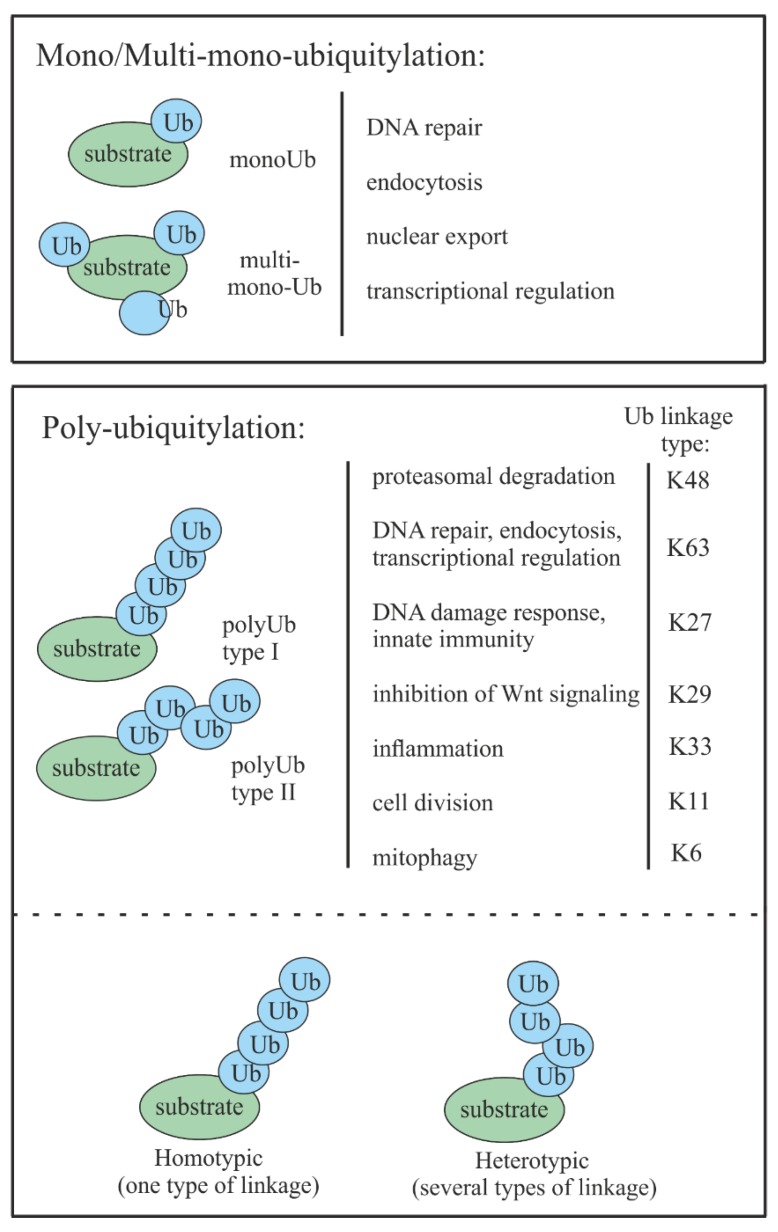
Schematic representation of different types of protein substrate Ub modifications together with their roles in cell functioning.

In addition to ubiquitylation types, a so-called ubiquitin code exists, which is defined by the specific position of a bond between ubiquitins [19]. It is known that ubiquitin has seven lysine residues, K6, K11, K27, K29, K33, K48, and K63. Each of these lysine residues as well as the primary amine at the N-terminus can be used as a site for the attachment of other ubiquitin molecules. The resulting polymers may contain multiple linkage types in mixed or branched topologies (heterotypic ubiquitin chains) or consist of ubiquitins connected by a single linkage type (homotypic ubiquitin chain). Naturally, the type and position of linkage defines the cell fate of the ubiquitin-tagged proteins [19] (Figure 2).

The best-known ubiquitin modification involves K48-linked polyubiquitin chains. This type of modification is commonly found in protein substrates tagged for proteasomal degradation [18]. Although K11-, K29-, and K63-linked chains have also been shown to be involved in proteasomal degradation, they appear to have a more regulatory role in a number of nonproteolytic processes [11], such as DNA repair, transcriptional regulation, or endocytosis [20,21,22]. While the knowledge about K6, K27, K29, and K33 linkages is limited, recent reports have suggested that K6-, K11-, and K33-linked poly- and monoubiquitylations regulate processes such as inflammation, cell division, and mitophagy, respectively [23,24,25].

## 3. Deubiquitinating Enzymes (DUBs)

The process of ubiquitylation is very dynamic and can be reversed by the action of specialized enzymes known as deubiquitinases (DUBs) [7].

**Figure 3:**
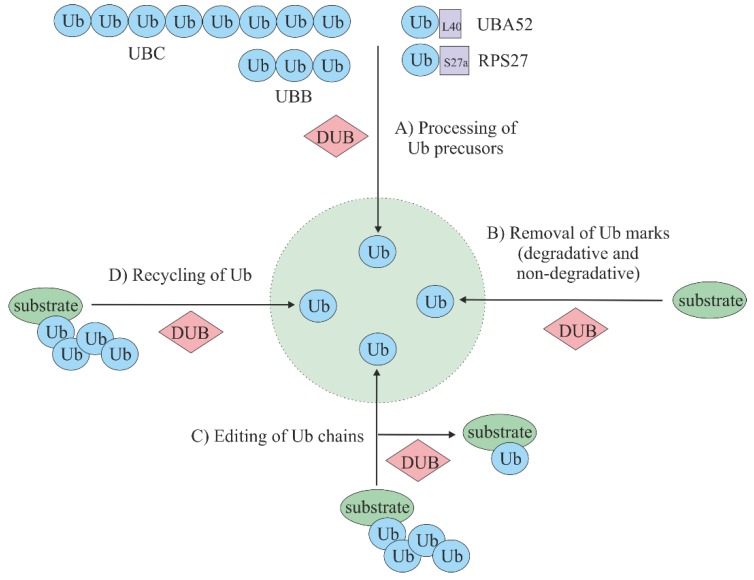
Cellular role of deubiquitinating enzymes (DUBs). (**A**) Processing of ubiquitin precursors. Ubiquitin is encoded by four genes: UBA52, RPS27, UBB, and UBC. In the case of UBA52 and RPS27, ubiquitin is produced as a precursor, where a single ubiquitin is attached to ribosomal proteins L40 or S27a. The UBC and UBB genes express precursors comprised of 3–10 single ubiquitins attached “head to tail”. The production of free ubiquitin out of the precursor forms is one of the main roles of DUBs. (**B**) Removal of degradative or nondegradative marks from protein substrates, therefore rescuing substrates from degradation or modulating ubiquitylation signaling. (**C**) Editing of ubiquitin chains by changing one type of ubiquitin signal to another (e.g., transformation of polyubiquitin tag to monoubiquitin tag). (**D**) Recycling that ensures ubiquitin re-enters the ubiquitin pool by preventing ubiquitin degradation in the proteasome or lysosome together with its substrate.

Overall, the general role of DUBs can be presented in four separate pathways that are all linked in order to maintain adequate ubiquitin homeostasis. One of the most important function of DUBs is (a) ubiquitin precursor processing and maturation. In addition, DUBs are capable of (b) removing ubiquitin marks from protein substrates, which can rescue proteins from degradation or modulate signaling. Further important functions of DUBs consist of (c) the editing of ubiquitin chains, which can help to convert one ubiquitin signal type to another; and (d) the recycling of ubiquitin, which ensures that ubiquitin re-enters the ubiquitin pool [26] (Figure 3).

About 100 DUBs are encoded by the human genome, and they are subdivided into six families according to their sequence and structural similarity: JAB1/MPN/MOV34 metallopeptidases (JAMMs), ubiquitin carboxy terminal hydrolases (UCHs), otubain/ovarian tumor proteases (OTUs), Machado–Joseph disease protein domain proteases (MJDs), the newly discovered motif interacting with Ub-containing novel DUB (MINDY) family, and ubiquitin-specific proteases (USPs). The latter class is considered the largest of the DUBs and includes approximately 60 proteases. All of the DUBs are cysteine proteases, except JAMMs, which belong to zinc metalloproteases [27,28]. DUB families that act as cysteine proteases rely on two–three amino acid residues (Cys, His, and optional Asn/Asp) in their active center, ensuring nucleophilic attacks on the isopeptide bond. On the contrary, JAMMs, which coordinate two zinc ions, use the ability to activate H_2_O in order to attack the isopeptide bond between ubiquitin and the target protein [26,29].

Taking into account emerging reports supporting DUB involvement in many disorders, including cancer, it is not a surprise that DUBs are becoming more and more attractive “druggable” targets [30]. Recent reports have shown that small-molecule DUB inhibitors targeting, e.g., USP14 and UCHL5, could be used as anticancer agents and are being tested in preclinical studies [31,32].

## 4. Hypoxia and Cancer

Many organisms have evolved adaptive mechanisms for survival under hypoxic conditions [33]. Hypoxia is also a very common stress phenomenon in solid tumors, where, due to increased cell proliferation, large tumor masses are formed. Cancer cells adapt to conditions with low oxygen availability through activating the formation of a tumor vasculature and metabolic reprogramming, including the activation of glycolysis and inhibition of mitochondrial function [5,34]. The activation of HIFs is usually the key event in this adaptation to low oxygen concentrations. HIFs exist as heterodimers composed of the hypoxia-inducible α-subunit (HIF-α) and the constitutively expressed β-subunit, also known as the arylhydrocarbon receptor nuclear translocator (ARNT) [35]. So far, three major members of the HIF α-family have been described: HIF-1α, HIF-2α, and HIF-3α [36,37,38]. From the three known HIF α-family members, HIF-1α is the best characterized. Initially, it was identified due to its sensitivity to low O_2_ levels, but it is now clear that HIF-1α can also be regulated by other factors, such as the activation or loss of tumor oncogenes or suppressors. There is evidence that has indicated HIF-1α accumulation even under normoxic conditions in tumor cells after the loss of tumor suppressors such as von Hippel-Lindau (VHL) or Phosphatase and Tensin homolog (PTEN) or the activation of oncogenes such as Rat sarcoma (RAS), sarcoma (SRC), and phosphoinositide 3-kinase (PI3K) [34,39].

HIF-1α is known to regulate the expression of more than 300 genes and noncoding RNAs [6,40], which control genomic stability, drug resistance, angiogenesis, vascular tone, glucose metabolism, cell proliferation, and survival. As a result, elevated levels of HIF-1α are often associated with a poor prognosis for cancer patients [41,42].

Although there have been emerging reports about the role of hypoxia, HIFs, and DUBs in cancer development, there is still a lack of knowledge about their reciprocal regulation.

## 5. Degradation of HIFs

An abundance of HIF α-subunits is mainly regulated on the post-translational level via its degradation [43,44,45,46,47], although mechanisms involving HIF transcription [34] and HIF-1α mRNA translation have also been described [35].

**Figure 4:**
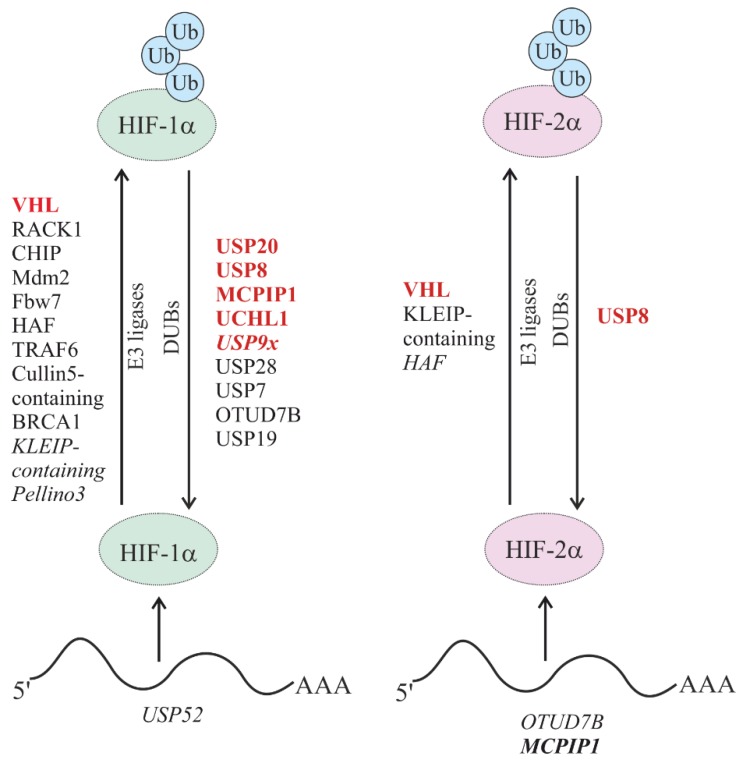
E3 Ub-ligating and deubiquitinating enzymes (DUBs) involved in hypoxia-inducible factor (HIF)-α stability and signaling. DUBs and E3 Ub ligases involved in the oxygen-dependent regulation of HIF-1α and HIF-2α degradation are depicted in red. DUBs and ubiquitin E3 ligases involved in oxygen-independent regulation of HIF degradation are depicted in black. DUBs involved in the regulation of HIF mRNA stability and/or expression are depicted in blue. Non-enzymatic and/or indirect regulations are indicated in italics.

The stability of HIF α-subunits is heavily reduced under normoxic conditions due to its rapid degradation via the proteasomal pathway [36], though lysosomal degradation can contribute to HIF-1α degradation during chaperone-mediated autophagy (CMA) [37]. In addition to hypoxic conditions, several signaling pathways have been shown to regulate the stability and transactivity of HIF-1α via post-translational modifications, including hydroxylation, acetylation, phosphorylation, SUMOylation, and ubiquitylation [38]. Therefore, it is not a surprise that several E3 ligases and DUBs can be involved in HIF α-subunit regulation (Figure 4).

### 5.1. Oxygen-Dependent Degradation of HIFs

**Figure 5:**
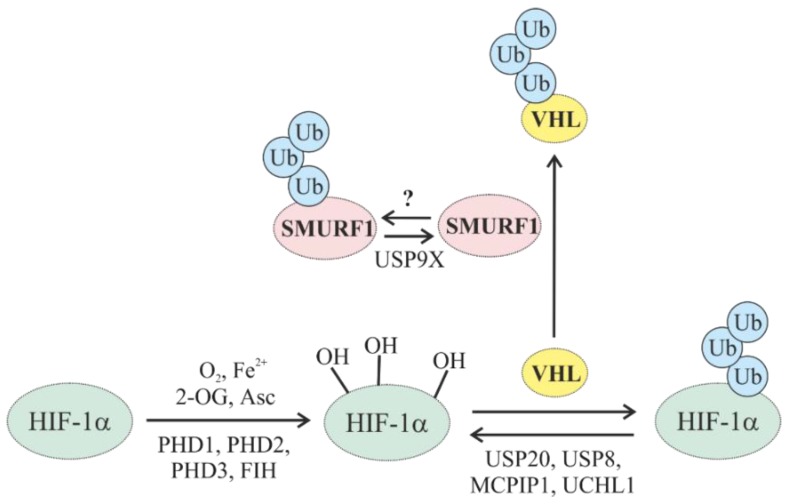
Involvement of E3 ligases and DUBs in the oxygen-dependent regulation of HIF. HIF-α subunits are hydroxylated in an O_2_-, Fe^2+^-, 2-oxoglutarate (2-OG)-, and ascorbate-dependent reaction. Hydroxylated HIF-α subunits are then recognized by von Hippel-Lindau (VHL), ubiquitylated, and degraded by the proteasome. VHL itself can be degraded by the E3 ligase SMURF1 (SMAD ubiquitination regulatory factor-1), which can be opposed by USP9X. VHL-mediated ubiquitylation can be antagonized by the DUBs USP20, USP8, MCPIP1, and UCHL1. E3 ligases are depicted in bold.

The protein stability of HIF-α is crucially regulated by the oxygen-dependent hydroxylation of two proline residues (P402 and P564 in human HIF-1α; P405 and P531 in human HIF-2α) located in the oxygen-dependent degradation domain (ODDD). The hydroxylation reactions are carried out by at least three HIF proline 4-hydroxylases known as proline hydroxylase domain-containing enzyme family members (PHD1–3) or *egl-9* (*Caenorhabditis elegans*) gene Homologs (EGLN1-3), which function as cellular O_2_ sensors [48]. The hydroxylation of the proline residues marks the HIF α-subunits for ubiquitylation through an E3 ligase complex containing the tumor suppressor protein von Hippel-Lindau (VHL) and for subsequent proteasomal degradation [49]. Moreover, another hydroxylation at an asparagine residue in the C-TAD of HIF α-subunits carried out by the factor-inhibiting HIF (FIH) alters their transactivity by preventing recruitment of the coactivator p300 [50]. Interestingly, VHL itself is subject to proteasomal degradation promoted by an E3 ligase, SMURF1, which is deubiquitylated by the DUB USP9x [51]. Several E3 ligases and DUBs have been reported to participate in the regulation of HIF-α degradation in an oxygen-dependent manner (Figure 5) (Table 1).

#### 5.1.1. Oxygen-Dependent Degradation of HIF by E3 Ub Ligases

So far, the tumor suppressor protein VHL is the major E3 ligase substrate recognition component in oxygen-dependent HIF-α degradation. In addition to VHL, the RING–E3 ligase complex also contains Ring Box 1 (RBX1), Cullin 2, and Elongin B and C [52]. Often, VHL is mutated in hemangioblastomas [53], pheochromocytomas [54], and clear-cell renal carcinomas [55], illustrating the importance of VHL in the pathogenesis of these diseases. Tumors lacking VHL are in general characterized by increased HIF-α levels and the expression of HIF target genes: Vascular endothelial growth factor (VEGF) and erythropoietin (EPO) [56,57]. These characteristics validate the correlation between HIF-α and VHL [49].

#### 5.1.2. Regulation of HIF Hydroxylases (PHDs and FIH) through Ubiquitylation

Even though HIF hydroxylase activity is mainly regulated via O_2_ availability, their abundance is also regulated by various E3 ligases catalyzing their ubiquitylation and consequent proteasomal degradation.

Although there are so far no known E3 ligases regulating PHD2, the protein stability and half-life of PHD2 are reported to be downregulated by its interactor peptidyl prolyl cis/trans isomerase FK506-binding protein 38 (FKBP38) [58].

The stability and abundance of PHD1 and PHD3 in the cell are regulated by the seven in absentia homolog (SIAH1/2) E3 ligases. Their activity increases under hypoxic conditions, resulting in less availability of PHD1/3 and HIF-1α stabilization [59]. The E3 ligase SIAH1 has also been reported to be a binding partner of the asparaginyl hydroxylase FIH, which is known as an oxygen-dependent repressor of HIF-1α, facilitating FIH degradation via the ubiquitin–proteasome pathway under hypoxic conditions [60]. Thus, SIAH ubiquitin ligases play a role as regulators of both types of HIF hydroxylases (PHDs and FIH) and represent an important part of feedback regulation.

In addition to SIAH1/2, the Cullin 3-based E3 ligase speckle-type POZ protein (SPOP) has been shown to regulate PHD1 via polyubiquitylation and proteasomal degradation. In line with this, it has been reported that elevated PHD1 levels and a loss of SPOP were linked to prostate cancer growth [61].

**Figure 6:**
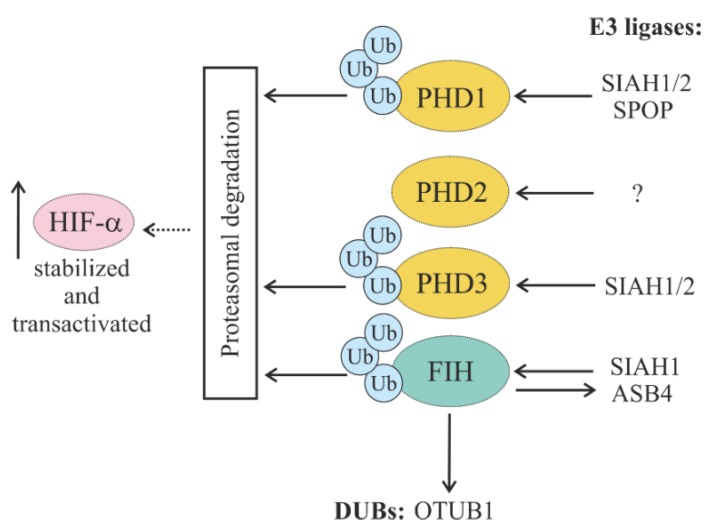
Schematic representation of HIF prolyl hydroxylases (PHDs) and factor-inhibiting HIF (FIH)) interplaying with E3 ligases and DUBs and their impact on HIF cellular abundance. Seven in absentia homologs ½ (SIAH1/2) and speckle-type POZ protein (SPOP) promote ubiquitylation of PHD1 and PHD3. So far, no E3 ligase is known for PHD2. Ankyrin repeat and SOCS box protein 4 (ASB4) and Otubain-1 (OTUB1) are hydroxylated by FIH.

Interestingly, a reciprocal regulation of E3 ligases by HIF hydroxylases has also been reported. Thereby, the ankyrin repeat and SOCS box protein 4 (ASB4), which is highly expressed in vascular tissue and serves as the substrate-recognizing protein of the SCF-like Elongin-Cullin-SOCS-box E3 ubiquitin–protein ligase complex, was shown to be hydroxylated by FIH, leading to vascular differentiation in an oxygen-dependent manner [62]. Together, the O_2_-dependent abundance of HIF-α transcription factors and their major hydroxylases is regulated by feedback cycles involving several E3 ligases (Figure 6).

#### 5.1.3. Regulation of Oxygen-Dependent HIF Degradation by DUBs

In the context of the oxygen-dependent HIF degradation pathway, there are only a few DUBs that appear to regulate, mainly HIF-1α.

The first described DUB that was shown to counteract VHL-mediated ubiquitylation of HIF-1α was USP20 (VDU2) [63]. According to those findings, USP20 binds to and stabilizes HIF-1α. As a consequence of this interaction, USP20 induces the expression of HIF-1α target genes, such as vascular endothelial growth factor (*VEGF*). On the other hand, the VHL-interacting deubiquitinase USP33, which has a strong homology with USP20 in its N-terminus and C-terminus and shares an approximately 59% identity with USP20, is not able to bind and stabilize HIF-1α [63]. Besides its involvement in hypoxia signaling, USP20 is also reported to be dysregulated in cancer, and its depletion is known to be associated with increased chromosomal aberrations, malignant transformation, and tumor growth [64]. Due to its ability to inhibit the malignant characteristics of gastric cancer cells via the positive regulation of claspin, USP20 is considered to play a tumor-suppressing role in gastric cancer [65]. Further, it has been shown that claspin expression in gastric cancer samples correlates with USP20 expression and that low claspin and USP20 levels are associated with worse overall survival [66]. However, these findings were contradicted by a recent report showing that USP20 regulates the deubiquitylation of β-catenin to control its stability, thereby inducing proliferation, invasion, migration, and chemoresistance in multiple cancer cells [67]. Thus, it remains open whether USP20 is a tumor suppressor or activator: Cell type-dependent effects are likely to be considered.

HIF-1α can also be deubiquitylated by the monocyte chemoattractant protein-1 (MCP-1)-induced protein-1 (MCPIP1). MCPIP1 is a zinc-finger protein with ribonuclease activity that is mainly crucial in the regulation of stability of transcripts related to inflammatory processes. This process appears to be rather important in vessel growth, as MCPIP causes HIF-1α deubiquitylation and nuclear localization and the induction of its target genes, such as Cyclooxygenase-2 (*COX-2*) and *VEGF* [68]. The MCPIP1–HIF axis also appears to be important in the protection of the liver against ischemia reperfusion injury [69] and the development of clear-cell renal carcinoma [70]. Moreover, the antidicer RNase activity of MCPIP is able to suppress the levels of miRs modulating HIF-1α and sirtuin-1 (SIRT-1) expression, which also contributes to angiogenesis activation [68].

MCPIP1 is also shown to affect HIF-2α at the transcript level, and HIF-2α in turn also regulates the expression of MCPIP1 [70]. Further, it has been demonstrated that MCPIP1 can function as a tumor suppressor, as it is able to induce the apoptosis of breast tumor cells by selectively enhancing the decay of mRNA necessary for the expression of antiapoptotic genes (Bcl2L1, Bcl2A1, RelB, Birc3, and Bcl3) [71]. Moreover, it has been found that MCPIP1 depletion increases cancer cell proliferation [70].

The deubiquitinase USP8 has been found to be another protein that can reverse the VHL-mediated degradation of HIF-1. The action of USP8 involves binding to the PER-ARNT-SIM (PAS) domain of HIF-1α and is linked to the maintenance of a basal HIF-1α level under normoxia, which is essential for rabaptin-5 expression and endosome trafficking-mediated ciliogenesis. Further, it was shown that USP8 also likely functions as a DUB for HIF-2α [72]. Apart from HIFs, USP8 is involved in epidermal growth factor receptor (EGFR) turnover, thus rescuing EGFR from lysosomal degradation [73], and mutations in the USP8 gene have been found in corticotroph adenomas, which could cause Cushing’s disease via activation of EGFR signaling [74].

The X-linked deubiquitinase USP9x was reported to affect the ubiquitylation status of HIF-1α indirectly by reducing VHL protein levels via the deubiquitylation of SMURF1, an E3 ligase targeting VHL [51]. USP9x, due to its ability to regulate SMAD family member 4 (SMAD4) and apoptosis signal-regulating kinase 1 (ASK1), is also involved in regulating cancer-associated transforming growth factor- β (TGF-β) [75] and mitogen-activated protein kinase (MAPK) signaling pathways [76]. Decreased levels of both USP9x mRNA and protein were reported to correlate with poor survival in patients with pancreatic ductal tumors, supporting its role as a tumor suppressor [77]. In addition, a correlation between the level of USP9x and the pro-survival-induced myeloid leukemia cell differentiation protein (MCL-1) was shown in follicular lymphomas and diffuse large B-cell lymphomas [78].

Ubiquitin carboxyl terminal hydrolase L1 (UCHL1) could abrogate VHL-mediated ubiquitylation of HIF-1α and promote metastasis in murine models of pulmonary metastasis [79]. Moreover, recent findings have shown that UCHL1 is subjected to oxidative carbonylation, which hampers its activity [80,81] and links its function to oxygen signaling. In addition, the levels of UCHL1 were shown to correlate with HIF-1α levels and to associate with a poor prognosis in patients with breast and lung cancer [79]. UCHL1 was also reported to be overexpressed in gastric cancer [82] and in myelomas [83], while it was silenced via methylation in several colon cancer cell lines [84], illustrating its potentially dual role in cancer development. Further, UCHL1-mediated HIF-1 dependence changed the antioxidant cellular status by increasing the intracellular glutathione levels, which promoted conversion of the cells into a radioresistant phenotype [85]. Interestingly, UCHL1 not only functions as a DUB, but in vitro, upon the formation of dimers, it was shown to act as a ubiquitin ligase [86].

Together, O_2_-dependent HIF degradation is regulated by a complex network of DUBs and E3 ligases that are on different levels of control and directly affect HIF ubiquitylation or indirectly affect HIF hydroxylases.

### 5.2. Oxygen-Independent Regulation of HIFs

**Figure 7:**
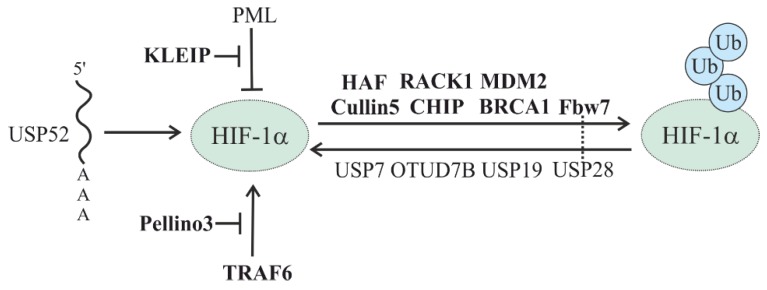
Involvement of E3 ligases and DUBs in the oxygen-independent regulation of HIF. E3 ligases are depicted in bold. The dotted line indicates known direct interactions between an E3 ligase and DUB.

Although the O_2_-dependent and VHL-mediated degradation system is the predominant one regulating HIF-α subunit stability, there are other O_2_-independent mechanisms that modulate their stability (Figure 7) (Table 2).

#### 5.2.1. HSP-Mediated HIF Degradation

One of the first proteins involved in O_2_-independent HIF degradation was the receptor for activated C kinase 1 (RACK1). RACK1, in concert with Elongin-C/B, was found to compete with heat shock protein 90 (HSP90) for binding to the HIF-1α PAS-A domain and to recruit Elongin-C/B and other components of the VHL E3 ubiquitin ligase complex to drive HIF-1α degradation [87]. In addition, the RING E3 ubiquitin ligase scaffold protein Cullin 5 was also shown to be involved in HSP90-mediated regulation of HIF-1α stability [88].

Further, the action of RACK1 could be inhibited by the calcium- and calmodulin-dependent serine/threonine phosphatase calcineurin. Thereby, the catalytic domain of calcineurin binds to RACK1 and dephosphorylates serine 146, which inhibits RACK1 dimerization and the recruitment of Elongin-C [89]. The mammalian septin family member SEPT9v1, a specific interactor of HIF-1α but not HIF-2α, was reported to negatively regulate this mechanism by preventing HIF-1α interaction with RACK1 [90]. A second negative regulator is the cleaved intracellular domain of the receptor-tyrosine kinase ERBB4, which also interacts with HIF-1α and inhibits RACK1-dependent HIF-1α degradation [91].

RACK1 complex-mediated HIF degradation can also be modulated by spermidine/spermine N-acetyltransferasees-1 and -2 (SSAT1, -2). While SSAT1 acts by stabilizing the interaction between HIF-1α and RACK1, SSAT2 stabilizes the interaction between VHL and Elongin-C. Thus, the paralogs SSAT1 and SSAT2 can play complementary roles in promoting HIF-1α degradation [92,93].

Heat shock protein 70 (HSP70), through recruiting the ubiquitin ligase C terminus of the HSC70-interacting protein (CHIP), promotes HIF-1α but not HIF-2α degradation via both proteasomal and autophagic machinery. The disruption of HSP70–CHIP interaction blocks HIF-1α degradation mediated by HSP70 and CHIP [46,94,95]. Moreover, CHIP was shown to be required for the chaperone-mediated degradation of HIF-1α by the lysosome. Thereby, the pentapeptide region of HIF-1α between N529 and L533 was reported to bind heat shock 70-kDa protein 8 (HSPA8) and lysosome-associated membrane protein 2A (LAMP2A), which recruited the K63-ubiquitylated HIF-1α to the lysosome [96,97].

#### 5.2.2. Other E3 Ligases in the Oxygen-Independent Degradation of HIF

Mouse double-minute 2 homolog proto-oncogene (MDM2) was reported to be implicated in HIF-1α regulation via both a direct [98] and p53-dependent mechanism [99]. In the case of the direct action, it was shown that MDM2 could regulate HIF-1α stability due to MDM2’s E3 ligase activity. Moreover, the action of MDM2 under hypoxia was controlled by the PTEN/PI3K/AKT signaling axis [98]. In the indirect mechanism, MDM2 was found to form a ternary p53/MDM2/HIF-1α complex, which is degraded in a p53-dependent manner. Similarly to p53, another tumor suppressor, TAp73, is thought to function as a scaffold that mediates the association between MDM2 and HIF-1α and its oxygen-independent proteasomal degradation [100].

Hypoxia-associated factor (HAF), which acts as a ubiquitin E3 ligase, is reported to play a dual role in the regulation of HIF-α stability. Thereby, it is supposed to mediate a switch from HIF-1α to HIF-2α-dependent transcription during tumor hypoxia by selectively degrading HIF-1α and promoting HIF-2α transactivation without affecting HIF-2α levels [101,102]. Additionally, the ability of HAF to activate HIF-2α-dependent transcription is dependent on hypoxia-induced HAF SUMOylation [103], thus adding another layer of complexity here.

In contrast to the view that the ubiquitylation of HIF is commonly associated with its degradation, the E3 ligase TNF receptor associated factor 6 (TRAF6) was reported to increase HIF-1α polylysine-63 ubiquitylation and to protect it from proteasomal degradation [104]. It was also reported that TRAF6-mediated monoubiquitylation and ATM-mediated phosphorylation of histone H2AX, which interacts with HIF-1α to prevent its degradation, promoted HIF-1α-driven tumorigenesis, glycolysis, and metastasis [105]. Interestingly, TRAF6-mediated HIF-1α stabilization can be switched off by the E3 Ub ligase Pellino3. Thereby, Pellino3 ubiquitylates TRAF6 with lysine 63-linked polyubiquitin chains to block its interaction with HIF-1α, resulting in reduced lysine 63-linked polyubiquitylation of HIF-1α, making it more prone to lysine 48-linked polyubiquitylation and proteasomal degradation [106].

Another Ub E3 ligase component having a positive effect on HIF is the BTB-kelch protein (KLEIP, also known as KLHL20). This HIF-1-induced protein functions as a substrate adaptor of Cullin 3-based ubiquitin ligases. Together with cyclin-dependent kinase 1/2 (CDK1/2) and peptidyl-prolyl cis-trans isomerase NIMA-interacting 1 (PIN1), KLEIP mediates proteasomal degradation of the tumor suppressor promyelocytic leukemia protein (PML). The reduced PML levels lead to a derepression of mammalian target of rapamycin (mTOR), which in turn participates in a feedback mechanism to amplify HIF-1α signaling, thus facilitating tumor progression [107]. Moreover, stabilization and mRNA expression of HIF-2α were found to be regulated by KLEIP. Despite the molecular details of KLEIP-dependent HIF-2α regulation being largely unclear, they appear not to be involved in cancer, but rather in the regulation of late-stage pulmonary maturation in mice [108].

An interaction between the tumor suppressor breast cancer type 1 susceptibility protein (BRCA1), which possesses ubiquitin E3 ligase activity, and HIF-1α was also found to be important in regulating HIF-1α stability in human breast cancer cells. However, so far it is unclear whether BRCA1-dependent ubiquitylation is involved in this process [109].

It is now well known that the phosphorylation of HIF-1α by glycogen synthase kinase-3 (GSK-3) is the signal for the recruitment of the E3 ubiquitin ligase F-box protein Fbw7. Similarly to VHL, the Fbw7-containing E3 ubiquitin ligase tags HIF-α subunits with ubiquitin, therefore promoting their proteasomal degradation [110,111,112].

#### 5.2.3. Oxygen-Independent Regulation of HIF Stability by DUBs

The above-mentioned Fbw7-mediated process of HIF-1α ubiquitylation is reversible and can be antagonized by USP28 [111]. This process affects hypoxia- and HIF-1α-dependent cell proliferation, colony formation, and angiogenesis [111]. Although the USP28-dependent regulation of HIFα is linked with tumorigenesis, USP28 is also known to stabilize oncoproteins such as c-MYC [113], c-JUN, and NOTCH [114]. Hence, the role of USP28 in tumorigenesis may vary depending on the tissues or cells involved. While reports have indicated that a lack of USP28 could reduce colon cancer in mice [114] and that USP28 could also be a prognostic marker in bladder [115] and gastric cancers [116], a lack of USP28 promoted liver cancer and correlated with a worse survival of patients with invasive ductal breast carcinoma: Mouse xenograft experiments with USP28-lacking breast cancer cells supported these findings [117].

USP7 (HAUSP), which is known to regulate p53 availability in the cell through its direct stabilization and/or indirectly by stabilizing MDM2 [118], was shown to have the ability to deubiquitylate HIF-1α under normoxia and to promote epithelial–mesenchymal transition and carcinogenesis [119]. Interestingly, hypoxia enhanced the function of USP7 via K63-linked polyubiquitylation at K443, the latter mediated by the E3 ubiquitin ligase HectH9 [119]. In addition, hypoxia-dependent USP7 induction was found to be associated with histone 3 lysine 56 (H3K56) acetylations that were mediated by the histone acetylase CREB-binding protein (CBP) [119].

Another DUB, USP19, which can affect the degradation of ER-associated degradation (ERAD) substrates [120], has also been proposed to function as a regulator in the hypoxia signaling pathway. This was based on findings showing that USP19 can interact with the bHLH-PAS domain of HIF-1α, but not HIF-2α, and with SIAH1 and SIAH2, which are known components of the hypoxia pathway since they regulate the presence of PHD 1/3 enzymes [121,122]. In the absence of USP19, HeLa cells fail to mount an appropriate response to hypoxia, indicating an important role of this enzyme in both normal and pathological conditions [123].

USP52 is another important regulator of HIF-1α, but it is active toward HIF-1α mRNA. This pseudo-DUB/deadenylase appeared to be a key component of the P-bodies required to prevent HIF-1α but not HIF-2α mRNA destabilization. Thereby, depletion of USP52 reduced the HIF-1α mRNA levels via a 3’-untranslated region-dependent but poly(A)-tail-length-independent interference mechanism, with the effect that HIF-1α-regulated hypoxic targets were expressed at a lower level [124].

The DUB OTU domain-containing protein 7B (OTUD7B or Cezanne) was reported to be of regulative importance and to protect HIF-1α and HIF-2α [125]. The regulation of OTUD7B expression in response to hypoxia itself seemed to be selective, with endothelial cells showing an induction of OTUD7B expression [126] and no OTUD7B response in HeLa or U2OS cells [125]. However, the knockdown of OTUD7B decreased HIF-1α and HIF-2α levels and increased K11 linkages on HIF-1α, but not K48 linkages. Further, the effects of OTUD7B on HIF-1α were not dependent on PHD-mediated HIF-1α hydroxylation, but required VHL. Interestingly, experiments interfering with both the function of the proteasome and lysosomal HIF-1α degradation have indicated that OTUD7B effects were mediated by chaperone-mediated lysosomal HIF-1α degradation [125]. Moreover, HIF-2α appeared to be differently regulated, since VHL inactivation was unable to rescue the OTUD7B-mediated reduction of HIF-2α. Indeed, OTUD7B was found to control the expression of HIF-2α mRNA via the transcription factor E2F1 binding to two sites in the *HIF2A* promoter. Moreover, OTUD7B was found to regulate E2F1 protein abundance and hence take part in maintaining its basal levels [127]. Overall, these data suggest that mitogenic signals can promote cell cycle progression, in particular under hypoxia, in an OTUD7B- and transcription factor E2F1-dependent manner via the regulation of HIF-2α expression.

## 6. Hypoxia: A Novel Regulator of DUBs

**Figure 8:**
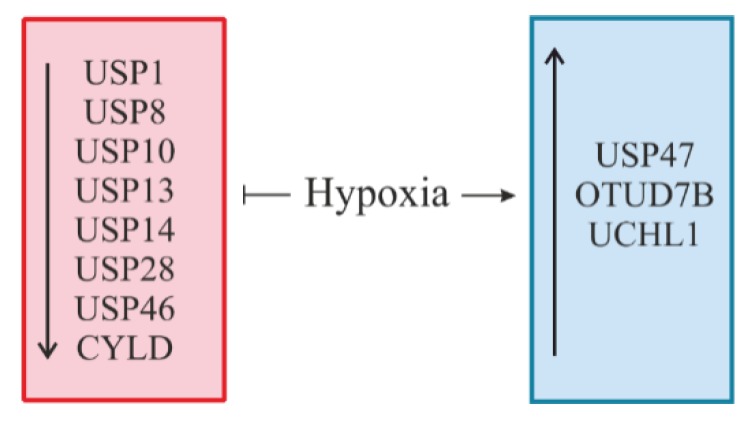
Regulation of DUBs by hypoxia. Hypoxia can cause either a decrease in the level of certain DUBs (depicted in red) or an increase in the level of other DUBs (depicted in blue).

As most of the important cellular enzymes, DUBs can be regulated on several different layers, including transcriptional regulation, mRNA stability and translation, protein stability, and catalytic activity. Despite increasing research about the function of different DUBs, knowledge about the regulation of DUBs in a cellular and physiological context is quite limited (Figure 8).

Taking into account that hypoxia is an indisputable cellular stressor, it is not unexpected that it is involved in the regulation of DUBs in order to adapt their functioning to the cell’s needs.

Although it cannot be precluded that hypoxia directly affects the activity or translation of DUBs, most regulation appears to take place at the mRNA level. It has been shown that exposure of human U87 glioma cells to hypoxia (3% O_2_) overnight diminishes mRNA levels of USP1, USP10, and USP14 [128]. USP1 is a negative regulator of DNA damage repair and deubiquitylates the monoubiquitylated Fanconi anemia group D2 protein (FANCD2) [129] and proliferating cell nuclear antigen (PCNA) [130]. USP10 is known to act as a tumor suppressor by stabilizing p53 and regulating MDM2-induced p53 nuclear export and degradation [131,132], and USP14 is required for the degradation of the chemokine receptor CXCR4 [133] and is able to inhibit endoplasmic reticulum-associated degradation (ERAD) via interaction with inositol-requiring enzyme-1α (IRE-1α) [134].

In addition, the expression of USP8, which has been shown to deubiquitylate HIF [72] and take part in EGFR turnover [73], was reported to be downregulated during intermittent hypoxia/reoxygenation conditions in renal tubular epithelial cells [135].

Further, decreased USP13 mRNA and protein expression could be detected after exposure of several melanoma cell lines to 2% O_2_ for 6 to 24 h. These changes appeared to be cell type and tissue-specific, since only melanoma cells (but not HEK293 or HeLa cells) displayed hypoxia-mediated USP13 reduction. Since USP13 limits the autodegradation of the E3 ubiquitin ligase SIAH2, decreased USP13 expression seen under hypoxia increased SIAH2 activity against its target substrates Sprouty2 and PHD3 [136]. In line with these results were findings where a depletion of USP13 in fibroblasts converted these fibroblasts into cells with a more aggressive phenotype with enhanced proliferative, migratory, and invasive capacities. Additionally, USP13 interacted with the tumor suppressor phosphatase and tensin homolog (PTEN). Downregulation of USP13 correlated with PTEN ubiquitylation and degradation in lung fibroblasts [137]. Hence, the hypoxia-induced decrease in USP13 expression may be pathogenically important during melanoma carcinogenesis and fibroblast conversion in idiopathic pulmonary fibrosis.

Hypoxia, as well as treatment with Ni compounds, contributed to the transcriptional repression of USP28 in A549 lung cancer cells via increased dimethylation of histone H3 lysine 9 at the USP28 promoter. Moreover, the authors showed that besides transcriptional regulation, a decrease in the USP28 protein level was also associated with increased protein degradation. In addition, a decrease in USP28 expression contributed to a higher level of c-MYC ubiquitylation and thus promoted degradation of this oncoprotein [138]. In contrast, the hypoxia-mediated USP28 decrease could not be observed in either MDA-MB-231 breast cancer cells or in mouse embryonic fibroblasts [117].

Further, an enhanced degradation of the PHLPP Ser/Thr protein phosphatase tumor suppressor appeared to be the result of reduced USP46 mRNA expression under hypoxic conditions in colon cancer cells. Although HIF-1α (but not HIF-2α) seemed to contribute to the reduction of PHLPP expression under hypoxia, it was mechanistically dependent on an mTOR-mediated decrease in protein translation. As a functional consequence of the hypoxia-reduced USP46 levels, the colon cancer cell lines SW480 and HCT116 showed increased chemotherapy resistance [139].

Interestingly, hypoxia-induced zinc finger protein SNAI1, together with hairy and enhancer of split-1 (HES1) (known as transcriptional repressors), might also be involved in the downregulation of another DUB, CYLD. This mechanism is likely to occur in hypoxia-mediated decreases in CYLD mRNA and protein levels in glioblastoma multiforme (GBM) [140]. CYLD overexpression was also shown to suppress the expression of various proinflammatory cytokines induced by TNF-α stimulation, suggesting that a hypoxia-mediated CYLD decrease could contribute to an increase in sensitivity to inflammatory stimuli in GBM cells. In line with this, it was also reported that hypoxia could promote an E6-dependent ubiquitylation and degradation of CYLD in HPV-infected cells, activating in turn the proinflammatory transcription factor NF-κB [141]. 

USP47, which is known to deubiquitylate β-catenin, thus increasing the proliferation of lung and prostate cancer cells [142], was reported to be upregulated under hypoxic conditions [143]. This upregulation, mediated by transcription factor SOX9, was found to promote the deubiquitylation of SNAI1 in three colorectal cancer cell lines, which boosted the endothelial-to-mesenchymal transition [143].

There is also evidence showing that hypoxia can induce OTUD7B via a p38 MAPK pathway [126] and UCHL1 expression involving a HIF-α-mediated mechanism [144]. It was reported that HIF-1α and HIF-2α promoted UCHL1 transcription by binding potential HRE sites within the *UCHL1* promoter, therefore inducing cell apoptosis in hypoxia-induced neuronal injury following neuronal hypoxic ischemic encephalopathy [144].

Recent reports have also revealed that oxygen-regulated HIF hydroxylases could have an effect on the stability and/or activity of certain DUBs. For instance, oxygen-regulated FIH was reported to conduct hydroxylation of its substrate, the DUB OTUB1. Although FIH-promoted hydroxylation does not affect OTUB1 stability, it likely regulates cellular energy metabolism, which is reflected by altered phosphorylation of the AMP-activated protein kinase α (AMPKα). The molecular mechanisms underpinning this alteration in AMPKα phosphorylation remains so far unknown [145]. Moreover, it has been shown that PHD1 (EGLN2) can hydroxylate the transcription factor FOXO3a and thereby prevent it from interaction with USP9x [146], demonstrating an indirect effect of prolyl hydroxylation on a deubiquitinating enzyme.

## 7. Conclusions and Perspectives

Taking into account that the HIF pathway is a master regulator of cellular adaptation to hypoxia, the fact that it is tightly controlled in healthy cells in order to avoid its inappropriate activation does not come as a surprise. HIF signaling pathway dysfunction is usually observed in many tumors and is likely to promote and/or follow tumor growth and cancer development. HIF abundance in the cell is more likely to be regulated on its protein level via Ub-mediated degradation controlled by a great number of E3 ligases and DUBs (Table 1 and Table 2). One of the possible mechanisms leading to an unbalanced HIF-α level is the activity of specific DUBs and/or E3 ubiquitin ligases, some of which are reciprocally regulated by hypoxia. However, the current knowledge represents just a basic understanding of the interaction of DUBs and HIFs with different signaling pathways involved in a variety of diseases (cancer in particular). At the same time, the lack of knowledge increases the demand to further unravel the role of E3 ligases and DUBs and the mechanisms linking their action with HIF signaling to better understand the pathogenesis of cancer and its future therapies.

## Figures and Tables

**Table 1:** E3 ligases and DUBs affecting the oxygen-dependent regulation of HIFs and their involvement in cancer.

Enzymes	Involvement in HIF Regulation	Involvement in Cancer	References
Ub E3 Ligases			
VHL-containing	Ubiquitylates hydroxylated HIF-α for proteasomal degradation	Mutates in hemangioblastomas, clear-cell renal carcinomas, and pheochromocytomas	[53,54,55,147]
SIAH 1/2	Ubiquitylate PHD1/3, leading to HIF-1α stabilization; SIAH1 facilitates FIH degradation via the proteasomal pathway	Tumor suppressors in breast, gastric, and liver cancers and laryngeal squamous cell carcinoma (SIAH1) Oncogenes in breast cancer (SIAH2) and hepatocellular carcinoma (SIAH1)	[60,148,149,150,151,152,153]
SPOP	Ubiquitylates PHD1, promoting its proteasomal degradation	Promotes clear-cell renal cell carcinoma development Reduced in nonsmall-cell lung cancer Tumor suppressor role in prostate cancer, frequently mutated	[61,154,155,156]
DUBs			
USP8	Reverses the VHL-mediated degradation of HIF-1α and HIF-2α	Mutated in corticotroph adenomas of Cushing’s disease Involved in gefitinib resistance of nonsmall-cell lung cancer Overexpressed in cervical squamous cell carcinoma	[72,74,135,157,158]
USP9X	Affects the ubiquitylation of HIF-1α indirectly by reducing VHL via deubiquitylation of the E3 ligase SMURF1, which targets VHL	Promotes human pancreatic cancer Overexpressed in gastric cancer and breast carcinomas; correlated with higher histologic grades of breast cancer Suppresses colorectal cancer development	[51,159,160,161,162]
USP20	Counteracts the VHL-mediated ubiquitylation of HIF-1α	Tumor suppressor role in gastric cancer Overexpressed in colon cancer, positively regulates tumorigenesis and chemoresistance via stabilizing β-catenin	[63,65,66,67]
MCPIP1	Deubiquitylates HIF-1α; suppresses the levels of HIF-1α and SIRT-1 miR repressors	Repressed in clear-cell renal cell carcinomas Promotes vascularization and metastasis in breast tumor cells	[68,69,70,71,163]
UCHL1	Abrogates VHL-mediated ubiquitylation of HIF-1α	Overexpressed in lung adenocarcinomas, gastric cancer, and myelomas Tumor suppressor in ovarian cancer cells, contributing to cisplatin resistance Silenced via methylation colon cancer	[79,82,83,84,164,165]

**Table 2:** E3 ligases and DUBs affecting the oxygen-independent regulation of HIFs and their involvement in cancer.

Enzymes	Involvement in HIF Regulation	Involvement in Cancer	References
Ub E3 Ligases			
RACK1	Competes with HSP90 for binding to HIF-1α to drive HIF-1α degradation	Overexpressed in nonsmall cell lung cancer, pulmonary adenocarcinoma, hepatocellular carcinoma, glioma, and breast cancer Reduced in gastric cancer	[87,166,167,168,169,170,171,172,173]
CHIP	Promotes HIF-1α but not HIF-2α degradation via both proteasomal or lysosomal machinery	Overexpressed in gallbladder carcinoma and esophageal and prostate cancer Reduced in breast, gastric, pancreatic, colorectal, and nonsmall cell lung cancer	[46,94,95,96,97,174,175,176,177,178,179,180,181]
MDM2	Regulates HIF-1α stability directly due to E3 ligase activity or indirectly by forming a ternary complex, which is degraded in a p53-dependent manner	Overexpressed in mesothelioma and in ovarian cancer	[98,99,182,183,184]
Fbw7	Recruited to GSK-3-phosphorylated HIF-1α for proteasomal degradation.	Highly mutated in cholangiocarcinomas and T-cell acute lymphocytic leukemia Reduced in glioma, pancreatic cancer Overexpression of FBW7 correlates with better survival of patients with colorectal cancer Regulates cell migration and angiogenesis in an HIF-1α-dependent manner	[110,111,112,185,186,187,188]
HAF	Selectively degrades HIF-1α and promotes HIF-2α transactivation during hypoxia	Associated with decreased progression-free survival in patients with clear-cell renal cell carcinoma Overexpression promotes GBM initiation and progression in mice Overexpressed in brain, breast, and colorectal cancer	[101,102,189,190,191]
TRAF6	Increases HIF-1α polylysine-63 ubiquitylation, protecting it from proteasomal degradation; TRAF6-ATM-H2AX signaling axis promotes HIF1α stabilization and activation	Predicts poor survival outcome in human breast, urothelial bladder, and gastric cancer patients Overexpressed in esophageal and pancreatic cancer and melanoma	[104,192,193,194,195,196]
Cullin 5-containing	Involved in HSP90-mediated regulation of HIF-1α stability	Reduced in breast and gastric cancer and in both endometrioid and serous endometrial adenocarcinomas Has antiproliferative effect in cervical and hepatocellular cancer	[88,197,198,199,200,201]
KLEIP-containing	By reducing levels of PML, leads to a activation of mTOR, which promotes HIF-1α signaling; stabilizes mRNA expression of HIF-2α	Promotes tumor angiogenesis and tumor growth that is overexpressed in human prostate cancer mice	[107,108]
BRCA1	Regulates stability of HIF-1α (NRM)	Highly mutated in breast and ovarian cancer	[109,202,203]
Pellino3	Ubiquitylates TRAF6 with polylysine-63 to block its interaction with HIF-1α, making it more prone to proteasomal degradation		[106]
DUBs			
USP7	Deubiquitylation of HIF-1α	Overexpressed in esophageal squamous cell carcinoma, cervical cancer, and hepatocellular carcinoma	[119,204,205,206]
USP19	Stabilizes HIF-1α and interacts with SIAH1/2 and PHD1/3 regulators	Regulates the proliferation of several prostate and breast cancer cell lines Reduced in human kidney renal clear-cell carcinoma	[121,122,207,208]
USP28	Counteracts Fbw7-mediated HIF-1α ubiquitylation	Depletion can reduce colon cancer in mice Overexpressed in bladder and gastric cancers Reduction promotes liver cancer and correlates with a worse survival of patients with invasive ductal breast carcinoma	[111,114,115,116,117]
USP52	Stabilizes HIF-1α mRNA	Overexpressed in breast carcinomas	[124,209]
OTUD7B	Stabilizes HIF-1α and E2F1 transcription factor to control the expression of HIF-2α mRNA; affects HIF-2α at the transcript level	Reduced expression in hepatocellular carcinoma patients and in lung adenocarcinoma High levels are associated with a good prognosis in patients with nonsmall cell lung cancer Suppresses Akt activation and Kras-driven lung tumorigenesis in mice	[70,125,127,210,211,212,213]
